# Assessment of the Readability of Web-Based Patient Education Material From Major Canadian Pediatric Associations: Cross-sectional Study

**DOI:** 10.2196/31820

**Published:** 2022-03-16

**Authors:** Alice Man, Courtney van Ballegooie

**Affiliations:** 1 Faculty of Medicine University of British Columbia Vancouver, BC Canada; 2 Department of Experimental Therapeutics British Columbia Cancer Research Institute Vancouver, BC Canada

**Keywords:** health literacy, accessibility, online health information, pediatrics, patient education

## Abstract

**Background:**

Web-based patient education materials (PEMs) are frequently written above the recommended reading level in North America. Poor PEM readability limits the accessibility of medical information for individuals with average literacy levels or lower. Pediatric hospital and association websites have not only been shown to be a preferred source of information among caregivers but have also become a necessity during the COVID-19 pandemic. The readability of Canadian pediatric association websites has not yet been assessed.

**Objective:**

The aim of this study is to determine if the content of PEMs from Canadian pediatric associations is written at a reading level that the majority of Canadians can understand.

**Methods:**

A total of 258 PEMs were extracted from 10 Canadian pediatric associations and evaluated for their reading level using 10 validated readability scales. The PEMs underwent a difficult word analysis and comparisons between PEMs from different associations were conducted.

**Results:**

Web-based PEMs were identified from 3 pediatric association websites, where the reading level (calculated as a grade level) was found to be an average of 8.8 (SD 1.8) for the Caring for Kids website, 9.5 (SD 2.2) for the Pediatric Endocrine Group website, and 13.1 (SD 2.1) for the Atlantic Pediatric Society website. The difficult word analysis identified that 19.9% (SD 6.6%) of words were unfamiliar, with 13.3% (SD 5.3%) and 31.9% (SD 6.1%) of words being considered complex (≥3 syllables) and long (≥6 letters), respectively.

**Conclusions:**

The web-based PEMs were found to be written above the recommended seventh-grade reading level for Canadians. Consideration should be made to create PEMs at an appropriate reading level for both patients and their caregivers to encourage health literacy and ultimately promote preventative health behaviors and improve child health outcomes.

## Introduction

The internet is a valued source of health care information for patients and caregivers worldwide [1,2]. Patients have been shown to rate web-based health information as one of the most useful health care resources, second only to direct communication from a physician or nurse [3]. The internet not only serves as a source of supplemental reading following a doctor’s visit but can also inform patients on best health practices and encourage them to seek medical treatment for symptoms. This is particularly relevant during the COVID-19 pandemic as patients are seeking information about unprecedented medical concerns with limited access to health care [4,5].

Pediatric hospital and association websites are preferred sources of health information by caregivers among internet resources [6]. It is especially important that these websites are accurate and accessible to prevent misinformation. If the content is too difficult to understand, caregivers and patients may resort to using less accurate internet sources. This may exacerbate disparities in health outcomes since individuals with higher health literacy will have greater functional access to health-related content than those with literary barriers. These disparities have been highlighted through multiple studies, where low caregiver health literacy was shown to be associated with poor preventative health behaviors, increased pediatric emergency department use, nonurgent visits, and poorer child health outcomes [7-9].

In Canada, the average proficiency in literacy corresponds approximately to an eighth- to ninth-grade reading level, where over 45% of Canadian adults have been shown to have low literacy skills [10,11]. The readability of educational material is recommended to be at least 2 or more grade levels below the average Canadian reading level to ensure comprehension [12]. Therefore, all patient-related material should be written at a maximum of a seventh-grade reading level. As health literacy commonly requires the use of a combination of prose literacy, document literacy, and/or numeracy skills, adults may have a harder time understanding health-related content than typical prose [13]. Even with adequate literacy skills, many caregivers still have difficulty understanding well-established health-related information in order to care for their infant [14]. Health-related reading materials should be further simplified to account for these additional challenges.

Pediatric health literacy has been explored globally, focusing on a variety of topics and subspecialties within pediatrics. Overall, studies in North America, France, Australia, the United Arab Emirates, Turkey, and Brazil have found that pediatric health information has been written above an acceptable reading level [15-20]. Topics have ranged from mental health, otolaryngology, orthopedics, oral health, oncology, and consent and discharge forms [21-27]. In Canada, although a variety of topics and subspecialties have been studied as they relate to health literacy, such as oncology, microtia and aural atresia, and emergency medicine [28-30], no study has evaluated the pediatric information developed by major pediatric associations and societies from multiple disciplines. This study aims to evaluate the reading level of the web-based Canadian pediatric patient education material (PEM) from pediatric associations and societies and to provide specific recommendations to improve readability.

## Methods

### Sample Collection

During May and June 2020, all internet-based PEMs were downloaded from the pediatric associations’ websites. A total of 10 national associations were identified and are listed in Table 1 along with the number of unique PEMs obtained from each association. The downloaded PEMs included materials describing any topic with intended use by parents, guardians, or children on the pediatric websites. Therefore, this excluded any material intended for health care providers. PDF files were manually converted to plain text for further analysis. Text sections containing nonmedical information such as page numbers, disclaimers, tables, diagrams, phone numbers, emails, and webpage navigation were removed from each of the PEMs before analysis.

**Table 1:** A list of the pediatric associations that provide patient education material and the number of documents obtained from each.

Canadian pediatric association	Documents obtained, n
Canadian Pediatric Society	205
Canadian Pediatric Endocrine Group	46
Atlantic Pediatric Society	7
Canadian Association of Pediatric Surgeons	0
Canadian Association of Child Neurology	0
Canadian Pediatric Cardiology Association	0
Canadian Pediatric Anesthesia Society	0
Canadian Association of Pediatric Nephrologists	0
Canadian Academy of Child and Adolescent Psychiatry	0
Canadian Association of Pediatric Ophthalmology	0

### Document Readability Analysis

A readability assessment was performed on the PEMs using the software package Readability Studio Professional Edition (version 2019.3; Oleander Software Ltd). The readability scales used to determine the reading level, which was reported as a grade level, of the PEMs included 8 numerical scales and 2 graphical scales. The 8 readability scales included the Degrees of Reading Power–grade equivalent test (DRP-GE); Flesch-Kincaid Grade Level (FK); Simple Measure of Gobbledygook index (SMOG); Coleman-Liau index (CLI); Gunning Fog index (GF); New Fog Count (NFC); New Dale-Chall readability formula (NDC); and Ford, Caylor, Sticht scale (FORCAST). The 2 graphical scales included the Raygor Readability Estimate Graph (RREG) and the Fry Readability Graph (FRG). These 10 scales are often used when assessing medical text and offer externally validated measures of readability [31-33].

**Figure 1:**
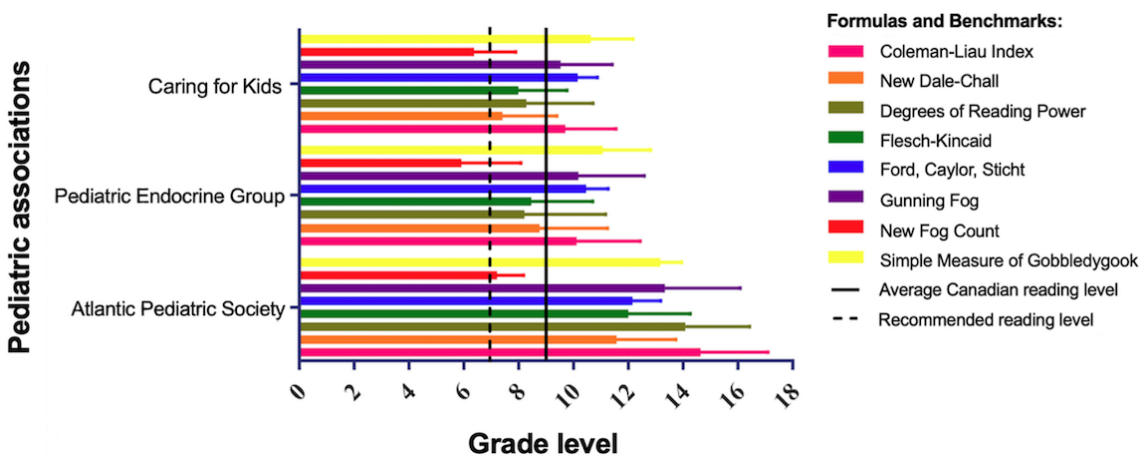
The reading level of patient education materials (PEMs) developed by the Atlantic Pediatric Society, Pediatric Endocrine Group, and Canadian Pediatric Society (Caring for Kids) as calculated by various numerical readability scales, compared to the average Canadian reading level and the recommended reading level for PEMs.

**Figure 2:**
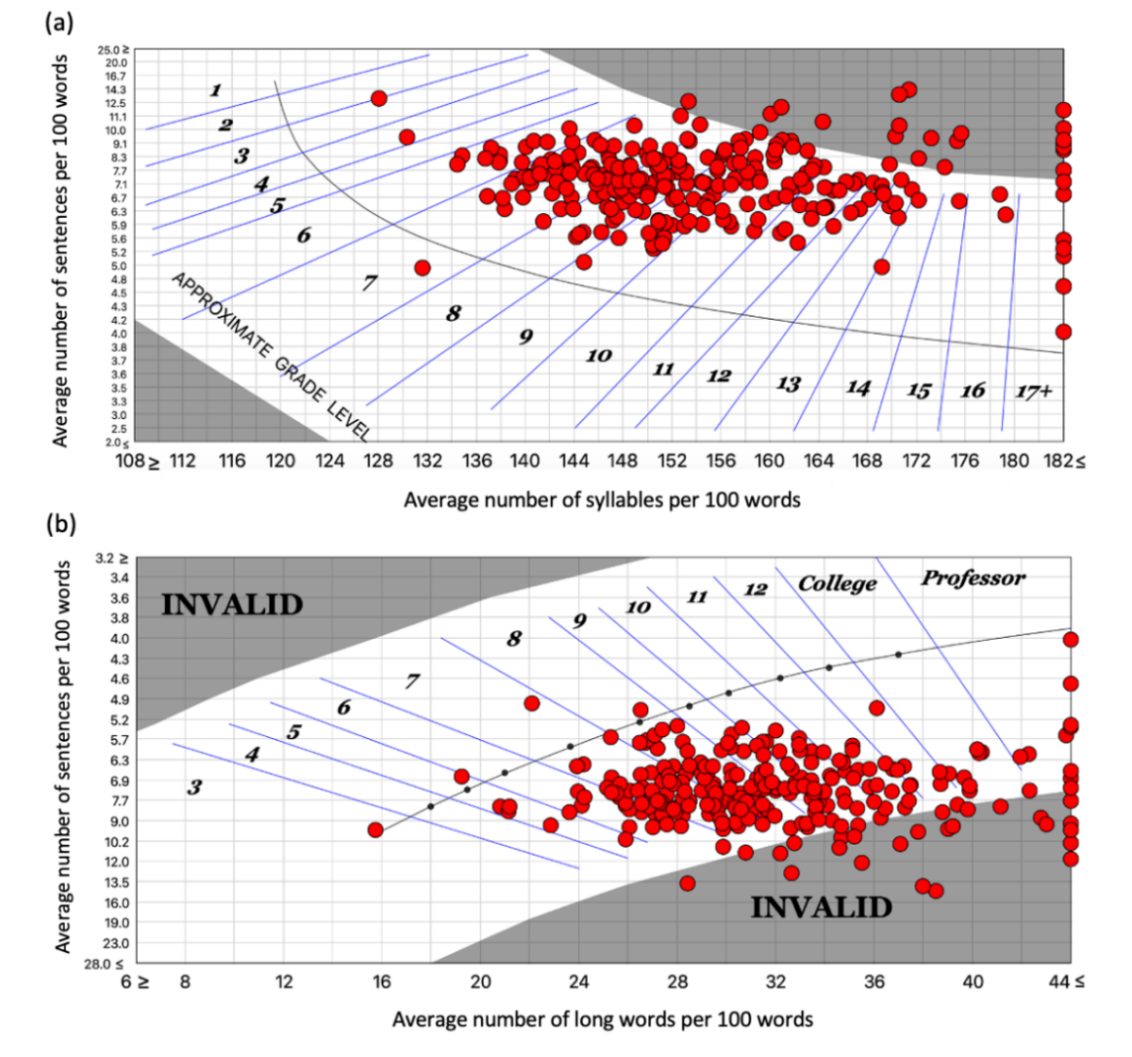
(A) The Fry Readability Graph of all high-sentence estimate web-based patient education materials (PEMs) collected from Canadian pediatric associations. The Fry Readability Graph visually demonstrates the readability of PEMs by the intersection of the number of syllables per 100 words and the number of sentences per 100 words. (B) The Raygor Readability Estimate Graph of all high-sentence estimate web-based PEMs collected from Canadian pediatric associations. The Raygor Readability Estimate Graph visually demonstrates the readability of the PEMs by the intersection of the number of long words per 100 words and sentences per 100 words. Within both graphs, the numbers within the graph indicate the approximate reading level (reported as a grade level) and the circles indicate reading levels of individual PEMs.

PEMs often contain text that must be modified before the analysis to appropriately apply the readability scales. This includes the removal of charts as well as the modification of bullet points to form complete sentences for analysis. To address the limitation of narrative-based readability scales, PEMs were ﻿individually edited to create high- and low-sentence documents, as performed by Perni et al [32]. For example, in high-sentence documents, each individual bullet point was treated as an independent sentence and resulted in a lower grade level estimate. On the other hand, low-sentence documents had each bullet point separated with a comma, with the final bullet point ending the sentence; this resulted in a higher grade level estimate [31,32]. The high- and low-level estimates were then averaged for further analysis. The associations’ readability level using the 8 numerical scales can be seen in Figure 1, and the 2 graphical scales can be seen in Figure 2.

### Difficult Word Analysis

A difficult word analysis was performed to identify the number and percentage of complex words (composed of 3 or more syllables), long words (composed of 6 or more letters), and unfamiliar words in each PEM according to the NDC criteria [34,35]. Once all the words were extracted from the PEMs, they were compared to the ﻿NDC word list as well as the New General Service List (NGSL). Words that appeared in either of the lists were removed and considered to be nonjargon words. All words that appeared in less than 3 PEMs were excluded from the analysis. Words with 3 or more syllables were then extracted, and the various tenses of the 10 most frequently identified words, where applicable, were combined. Alternative words were then proposed for any 3-syllable word that appeared in 3 or more PEMs, either using the Readability Studio Software, the Merriam-Webster thesaurus, or in consultation with a physician, to identify synonyms that can decrease the difficulty of the word.

### Statistical Methods

Graphical data in Figure 1 were reported as the arithmetic means with the error bars representing the standard deviations. Data sets had their normality tested using a Shapiro-Wilk test when central limit theorem conditions were not met. Equal variance was tested using a Brown-Forsythe test to determine if the data would need to be transformed before analysis. Normally distributed data with equal variance then underwent a one-way analysis of variance (ANOVA). If the data were not normally distributed, then a nonparametric Kruskal-Wallis test was employed. Multiple comparisons tests, such as Tukey tests, were used to identify differences between sample means in the ANOVA analysis [36]. The data were analyzed using Graph Pad Prism (version 9; GraphPad Software Inc).

## Results

### Document Readability Analysis

Following conversion to plain text, high- and low-sentence PEMs were subjected to 8 readability tests, including the DRP-GE, FK, SMOG, CLI, GF, NFC, NDC, and FORCAST. Figure 1 illustrates a summary of the results for the pediatric associations. The reading levels (reported as grade levels) measured by the 8 readability scales were averaged for each pediatric association, where the mean and standard deviations are reported as follows: Caring for Kids (8.8, SD 1.8), Pediatric Endocrine Group (9.5, SD 2.2), and the Atlantic Pediatric Society (13.1, SD 2.1). The overall mean was 9.1 (SD 2.4), with a grade-level range of 5 to 17. Note that Caring for Kids is a website developed by the Canadian Pediatric Society.

When the 8 readability scores of the individual PEMs were averaged, only 18 (7%) and 144 (55.8%) of the 258 PEMs, were below a seventh-grade and ninth-grade level, respectively. The RREG score of the high-sentence PEMs (Figure 2) ranges from a third-grade reading level to a grade level equivalent to that in university, with 26 (10.1%) and 127 (49.2%) of the 258 PEMs written at a grade level below 7 and 9, respectively. The FRG score of the high-sentence estimate, as seen in Figure 2, ranges from a third-grade to a 17th-grade (university-educated) reading level, with 14 (5.4%) and 118 (45.7%) of the 258 PEMs written at a grade level below 7 and 9, respectively.

The grade levels calculated by all 8 scales from the Atlantic Pediatric Society’s PEMs were also compared to those from Caring for Kids and the Pediatric Endocrine Group. Comparisons with 7 out of 8 reading tests were found to be statistically significant for both pediatric associations, where the NFC test was the only test to show no statistical significance (Table 2).

**Table 2:** Comparison of the reading level scores calculated by 8 readability scales of patient education materials (PEMs) from the Atlantic Pediatric Society (APS) with 2 other pediatric associations' PEMs.

Readability test	*P* value across all PEMs	*P* value for the pairwise comparison of the APS^a^ PEMs^b^ to other pediatric associations' PEMs^c^	
		Caring for Kids	Pediatric Endocrine Group
CLI^d^	<.001	<.001	<.001
NDC^e^	<.001	<.001	.02
DRP-GE^f^	<.001	<.001	<.001
FK^g^	<.001	<.001	.001
FORCAST^h^	<.001	<.001	<.001
GF^i^	<.001	<.001	<.001
NFC^j^	.08	.38	.13
SMOG^k^	<.001	.003	<.001

^a^APS: Atlantic Pediatric Society.

^b^PEM: patient education material.

^c^*P* values for comparisons across the different pediatric associations’ PEMs were calculated using analysis of variance (ANOVA) with a Welch correction nonparametric equivalent when applicable. *P* values for pairwise comparisons between ﻿PEMs were calculated using the Tukey, Tamhane, and Dunnett test.

^d^CLI: Coleman-Liau index.

^e^NDC: New Dale-Chall readability formula.

^f^DRP-GE: Degrees of Reading Power–grade equivalent test.

^g^FK: Flesch-Kincaid Grade Level.

^h^FORCAST: Ford, Caylor, Sticht scale.

^i^GF: Gunning Fog index.

^j^NFC: New Fog Count.

^k^SMOG: Simple Measure of Gobbledygook index.

### Difficult Word Analysis

From the difficult word analysis, it was determined that of all the words found in the PEMs, on average, 13.3% (SD 5.3%) were complex words which contained 3 or more syllables, 31.9% (SD 6.1%) contained 6 or more letters, and 19.9% (SD 6.6%) were unfamiliar words. All PEMs collected had a target audience of caregivers or pediatric patients (described as patients between the ages of 0 and 19). The most frequent terms included cannabis, marijuana, medication(-s), calcium, cortisol, and hepatitis. Table 3 describes the most frequent difficult words in compliance with the criteria described in the methods section.

**Table 3:** Difficult words found in the patient education materials analyzed, with alternative word recommendations.

Organization	Difficult word^a^	Frequency	Alternatives^b^
Atlantic Pediatric Society	Pediatric(-s), Pediatrician(-s)	41	Doctor for kids, doctor
	Adolescent(-s), Adolescence	7	Kids, children, teenage
	Developmental	4	Growth, stage, other abled
Pediatric Endocrine Group	Calcium	226	N/A
	Cortisol	180	Stress hormone
	Puberty, Pubertal, Puberties	161	N/A
	Genital(-s, -ia)	148	Private parts
	Injection(-s), Injectable	148	Shot
	Activate, Activated, Activating	125	Turn on, start, trigger
	Adrenal(-s)	102	N/A
	Vitamin	90	N/A
	Medication(-s)	90	Treatment, drug
	Pituitary	85	Brain gland
Caring for Kids	Cannabis, Marijuana	362	CBD^c^, THC^d^
	Medication(-s)	151	Treatment, drug
	Hepatitis	143	N/A
	Media	137	Online, T.V.^e^, print
	Diabetes	126	High sugars
	Vaccination(-s), Vaccinated	119	Shot
	Influenza	103	Flu
	Vitamin(-s)	101	N/A
	Breastfeeding	99	Nursing, feeding
	Pediatric, Pediatrician(-s)	97	Doctor for kids, doctor

^a^The following inclusion criteria were used for identifying a difficult word: (1) any word with ≥3 syllables that was used at least once in ≥3 patient education materials and (2) was unlisted on either the New Dale-Chall list of familiar words or the New General Service List.

^b^Alternatives selected are those that are considered synonymous and that decrease the individual word’s syllables and/or letter count.

^c^CBD: cannabidiol.

^d^THC: tetrahydrocannabinol.

^e^T.V.: television.

## Discussion

### Principal Findings

PEMs found on pediatric associations’ websites serve as an important link between health care professionals and caregivers. Through these web-based resources, parents can access reputable information endorsed by health care professionals to inform childcare practices on a day-to-day basis [1-3]. Although these resources are readily available with internet access, they are not always functionally accessible to all caregivers and patients. Pediatric PEMs have consistently been shown globally to be written at higher reading levels than recommended for a public audience, which is consistent with this study’s findings [9,15-20].

Based on the analyses using the DRP-GE, FK, SMOG, CLI, GF, NFC, NDC, and FORCAST scales, PEMs available on Canadian pediatric association websites were found to be written at a ninth-grade reading level (mean 9.1, SD 2.4) on average, wherein only 7% (18/258) of PEMs were written below the recommended seventh-grade reading level. Similar results were shown by the RREG and FRG (Figure 2), wherein only 5.4% (14/258) to 10.1% (26/258) of PEMs were found to be written below a seventh-grade reading level. This suggests that the PEMs cannot be easily understood by most Canadians, and even less so by pediatric patients. This is particularly true for the Atlantic Pediatric Society’s PEMs, which are written at a university reading level (mean 13.1, SD 2.1). In Table 2, it can be seen that the Atlantic Pediatric Society’s PEMs were statistically significantly different from other pediatric associations’ PEMs in a majority of the readability tests employed. This suggests that the Atlantic Pediatric Society should consider all parameters used in each readability test, such as word and sentence length, the number of syllables in each word, and the familiarity of the words used, to improve readability. In addition to reducing the reading level of the text directed to caregivers, pediatric associations should consider stratifying their websites to include simpler educational materials directed to children.

Although the study itself uses 8 numerical and 2 graphical readability indices to better represent the many parameters that are factored into readability, special emphasis should be given toward readability formulas designed for health care materials. This includes the SMOG and FRG, with the SMOG considered as the gold standard by many large institutes such as the National Cancer Institute [37]. When factoring this into account, the average reading level of the PEMs would be closer to an 11th-grade rather than a ninth-grade reading level. Although this study focused on PEMs derived from only Canadian pediatric hospitals and associations, patients retrieve information from a variety of other sources. Health content obtained from common sources, such as Wikipedia and other popular websites containing health information, has also been shown to be written at a reading level far above the seventh grade [38-40]. These findings suggest that even if pediatric patients use education materials outside of those analyzed in this study, they may still face challenges in identifying information that is at an appropriate reading level.

The difficult word analysis revealed long words were the most common type of difficult word in the PEMs. This is in line with another recent Canadian publication on cancer-related PEMs [34]. Difficult terminology should be replaced with more familiar alternatives whenever possible to improve the ease of understanding. For example, “beneficial” could be replaced with “helpful,” and “clinician” could be replaced with “doctor” for simplicity. Although the substitution of terminology is ideal, it may not be applicable to cases in which information must be fully and accurately communicated. In these situations, a clear definition should be included when the word is introduced. Although the content of the PEMs was analyzed for reading level and word difficulty in this study, additional factors such as organization, layout, and design can impact PEMs’ readability. Therefore, further studies should be undertaken once additional instruments, such as the Suitability Assessment of Materials and PMOSE/IKIRSCH document readability formula, are validated for medical literature [41,42].

### Limitations

The readability tests used in this study consider parameters such as word and sentence length, number of syllables per word, words per sentence, and the difficulty level of words [31-33]. Although the use of multiple tests allows for more dimensions of readability to be considered, there are still limitations to using readability tests overall. Tests that assess syllable count may overestimate the readability of the text. Monosyllabic medical terminology, such as the word “stent,” contributes to a lower readability score, but that may not necessarily reflect a person’s ability to understand the terminology [15,31]. Conversely, tests that assess word familiarity may underestimate the readability of the text. Well-known medical terminology, such as the word “pediatrician,” acts to increase readability scores but may not contribute to increased difficulty in understanding the text. Furthermore, the act of defining difficult words within the text, which would greatly improve comprehension, is also not considered to impact readability. Additionally, the readability tests in this study do not account for the formatting of the text or the inclusion of diagrams. Although bullet points were analyzed as both sentences and comma-separated phrases, this does not fully capture the improvement to readability that lists provide. The evaluation of communication tools such as tables and images should be considered for future studies, as they can also serve to improve the ease of understanding [12]. Lastly, interpretations of the results must be taken into context as only Canadian pediatric associations were assessed, with just 3 of the 10 associations having PEMs on their websites. The results are therefore not representative of the totality of the information that caregivers and pediatric patients would be exposed to.

### Conclusions

Overall, web-based PEMs developed by Canadian pediatric associations exceed the recommended seventh-grade reading level. Difficult words should be replaced when possible or defined, and educational content directed specifically toward pediatric patients should be included. Additional consideration should be placed on the incorporation of multimedia PEMs [43]. Qualitative studies should be conducted in the future to better understand caregiver and provider information needs, as well as the barriers toward implementing more functionally accessible PEMs on pediatric association websites [44-46]. Additionally, the quality of the PEMs should be evaluated to determine if the information provided is accurate [47-49]. Once collected, this data can be used to inform changes that improve the usefulness, quality, and accessibility of pediatric PEMs. As the role of technology in health care increases, it is important that all individuals are able to understand and use reputable resources on the internet.

## Abbreviations

ANOVA: analysis of variance
CLI: Coleman-Liau index
DRP-GE: Degrees of Reading Power–grade equivalent
FK: Flesch-Kincaid Grade Level
FORCAST: Ford, Caylor, Sticht scale
FRG: Fry Readability Graph
GF: Gunning Fog index
NDC: New Dale-Chall readability formula
NFC: New Fog Count
NGSL: New general service list
PEM: Patient education material
RREG: Raygor Readability Estimate Graph
SMOG: Simple Measure of Gobbledygook index
